# Quantitative analysis of upright standing in adults with late-onset Pompe disease

**DOI:** 10.1038/srep37040

**Published:** 2016-11-15

**Authors:** Maria Stella Valle, Antonino Casabona, Agata Fiumara, Dora Castiglione, Giovanni Sorge, Matteo Cioni

**Affiliations:** 1 Gait and Posture Analysis Laboratory, Department of Biomedical and Biotechnological Sciences, University of Catania, Catania, Italy; 2 Regional Referral Center for Metabolic Diseases, Pediatric Clinic, Department of Clinical and Experimental Medicine, University of Catania, Italy

## Abstract

Pompe disease is a rare disorder producing muscle weakness and progressive impairments in performing daily motor activities, such as walking and standing. Most studies have focused on dysfunctions at cellular level, restricting the examination of gross motor functions to qualitative or subjective rating scales evaluations. With the aim of providing an instrumented quantification of upright standing in Pompe disease, we used a force platform to measure the center of pressure over three foot positions and with eyes open and closed. Amplitude and variability of body sway were measured to determine the level of postural stability, while power spectrum analysis and nonlinear computations were performed to explore the structure of the postural control. In comparison with healthy participants, patients with Pompe disease showed a reduced level of postural stability, but irrelevant variations in frequency content and spatio-temporal structure of the sway motion were detected. Changes in foot position did not increase the postural instability associated with Pompe disease, but prominent worsening occurred in the patients when they stand with eyes closed, particularly along the anterior-posterior direction. These results provide objective elements to monitor deficiencies of upright standing in Pompe disease, emphasizing the specific contributions of sway direction and sensory deficits.

Pompe disease (PD) is a very rare disorder caused by mutations of the glucosidase alpha acid gene. These mutations reduce considerably the activity of the acid alpha-glucosidase enzyme, preventing an appropriate breakdown of glycogen to glucose in the lysosomes^1^,^2^,^3^.

Accumulation of lysosomal glycogen may potentially affect all tissues, but the most prominent effects are observable in muscle. In particular, the release of autolytic enzymes from collapsed lysosomes produces an extensive fibers and contractile apparatus degeneration, leading to progressive muscle weakness. However, the clinical spectrum of PD is very heterogeneous, exhibiting a variability with respect to the level of severity, the rate of disease progression, the extent of organs involved, and the age of onset^1^,^2^,^3^,^4^,^5^,^6^,^7^. Based on the age of onset of clinical symptoms, the diversity of phenotypes may be categorized in infantile and late-onset forms^1^,^2^.

Typically, in late-onset PD, the clinical symptoms may develop between the first and sixth decade, with muscle weakness localized mainly in paraspinal and proximal lower limbs muscles^1^,^3^,^8^,^9^. The diaphragm is also compromised, while the heart is generally spared^1^,^2^,^7^,^10^. The deficiency of respiratory function progressively increases, sometimes requiring ventilatory support^7^,^10^,^11^.

The muscle weakness and respiratory deficits may in part origin from degenerative nervous system processes. In fact, the presence of substantial glycogen storage has been reported in the peripheral and central nervous system^4^,^5^,^7^,^12^,^13^,^14^,^15^,^16^,^17^. In particular, spinal motoneurons lesions may impair motor units recruitment and spinal reflexes, reducing muscle strength^12^,^13^,^14^,^17^. In the same line, respiratory neurons dysfunctions may alter the activation and synchronization of the diaphragm with intercostal and accessory respiratory muscles^7^,^10^,^16^. Peripheral elements of the sensory or motor system are also affected, producing somatosensory and neuromuscular transmission deficits^6^,^12^,^13^,^15^,^17^. Most of the nervous degenerations progressively appear later in adults with PD^5^,^7^,^10^,^17^.

The functional consequences of this clinical spectrum may lead to important impairments in executing essential everyday motor abilities, such as standing posture and walking. For example, the weakness of trunk and lower limb muscles and the severe alteration of ventilation movements potentially worsen the maintenance of balance, increasing the risk of falls^18^,^19^,^20^.

Despite the important impact of PD on the basic motor abilities, most of experimental studies have focused on molecular and metabolic disorders or on dysfunctions of single organs or systems. To date, muscular strength and endurance capability have been the most examined motor functions^21^,^22^, while the deficits of gross motor abilities associated with PD have been reported in the form of qualitative information or subjective rating scales^22^,^23^,^24^.

Instrumented quantification of essential motor activities in persons with PD would provide more objective and valuable information on the severity of the motor dysfunctions and thus would help to monitor the disease evolution in natural condition and under therapeutic treatment.

While McIntosh *et al.*^25^ reported a detailed spatio-temporal description of the gait ability in PD, yet no authors have carried out objective measurements of upright standing in patients with PD.

Following the above line of thought, the main goal of this study was to provide an objective, instrumented quantification of the bipedal standing stability in adult patients with late-onset PD. To accomplish this purpose we addressed the following issues.

First, we compared the level of postural stability during quiet standing in patients with PD and healthy people, measuring the standard spatial and temporal parameters (stability-related parameters) of the center of pressure (COP). Based on clinical data, the most straightforward prediction is that the patients should exhibit larger extension and temporal variability of the COP displacement with respect to the healthy persons. However, the contribution of the anterior-posterior (AP) and medial-lateral (ML) components of the COP to the overall stability might show some specificity in persons with PD. In fact, during quiet stance, the oscillations in the ML direction are attenuated by passive anatomical constraints at ankle and knee joints, while the AP direction is controlled most entirely by active muscle contractions. Therefore, the muscle weakness in PD would impair mainly the postural stability along the AP direction.

Second, to test the adaptation of the static upright standing in patients with PD, postural changes associated with variations of foot position and visual condition were evaluated. During quite stance, healthy subjects respond to changes in foot configuration or vision condition with a moderate decrease of postural stability. Since the deficits associated with PD involve also the nervous system, we hypothesize that the patients should show more postural difficulties to face biomechanical and/or sensory changes when compared with the healthy subjects. Alternatively, the lack of differences between the two groups would be a sign of functional compensation for the muscle weakness.

Finally, in addition to the standard posturography measurements, we explored also the frequency domain and the nonlinear dynamic structure of the spatio-temporal signals produced by the COP motion (structure-related parameters). These analyses capture the level of complexity that is not detected by traditional measures of postural performance and may indicate possible changes in the patterns of controlling upright standing. Muscle weakness or sensory-motor deficits may induce a reorganization of these patterns, but, particularly in the adult form of PD, it is possible that well-consolidated postural schemes prevent adaptive variations.

## Results

Clinical data from five patients are summarized in Table 1. The evaluation of muscle strength for each participant was accomplished by using the Medical Research Council scale (MRC).

**Figure 1:**
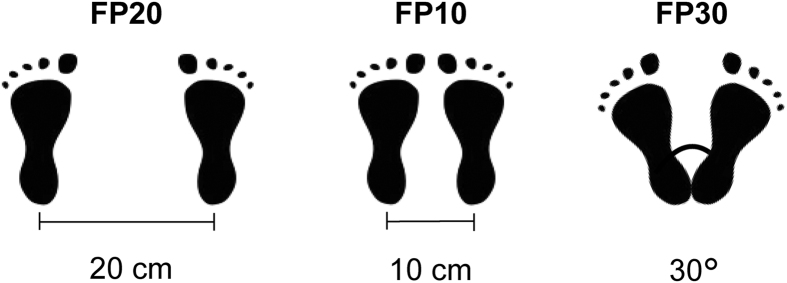
Representation of the foot positions. The schematic illustration depicts the three configurations of the foot position used in the experimental design. FP20, parallel feet with the heels spaced 20 cm; FP10, parallel feet with the heels spaced 10 cm; FP30, feet extra-rotated with the heels together and opening angle of 30°

The participants experienced standing posture over three foot positions (Fig. 1) and with eyes open and closed. The effects of group, foot position and vision condition on the stability- and structure-related parameters were analyzed by a three-way repeated measures Analysis of Variance (ANOVA), having group (patients vs controls) as between-subjects factor and foot position (FP10 vs FP20 vs FP30) and vision condition (eyes open vs eyes closed) as within-subjects factors.

The analysis of differences between the AP and ML directions for each two-dimensional parameter was modelled as a four-way repeated measures ANOVA with group as between-subjects factor, and foot position, vision condition, and direction as within-subjects factors.

### Analysis of the overall stability in keeping upright standing posture

**Figure 2:**
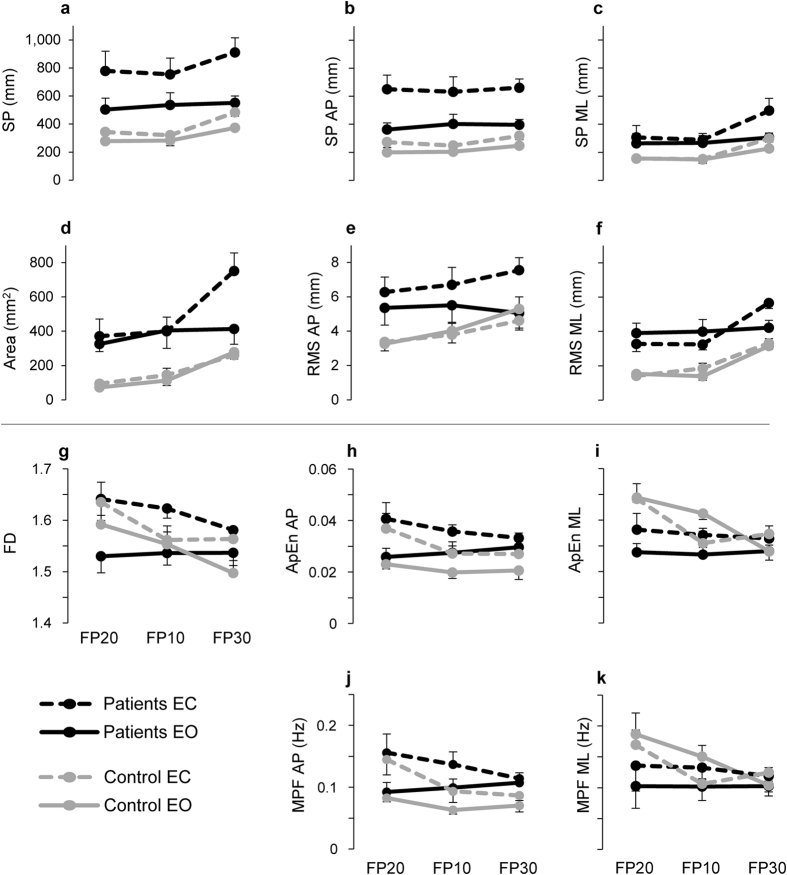
Quantitative changes in postural parameters between patients with Pompe disease and control group. The values of stability-related (**a–f**) and structure-related (**g–k**) parameters are reported as changes across the foot positions (FP20, FP10, FP30), and passing from eyes open (EO) to eyes closed (EC). Data are expressed as mean and standard error.

The majority of stability-related parameters were sensible in differentiating the patients from the healthy participants (Table 2: column 1, rows 1–6; Fig. 2a–f). In fact, except for the Sway Path (SP) measured along ML direction (SP ML), there were significant main effects of group for the SP, SP measured along AP direction (SP AP), Area, Root Mean Square (RMS) along AP and ML directions (RMS AP and RMS ML). For each parameter, the group of patients showed higher values than the group of control, regardless of foot position or vision condition (Table 3: rows 1–6 and 12–17; Fig. 2a–f).

The analysis of COP displacement along the AP and ML directions reveals a main significant effect of direction for the SP (F_1,8_ = 23.19, P = 0.001, 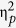
* = *0.74) and for the RMS (F_1,8_ = 34.95, P < 0.001, 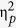
* = *0.81), with the AP direction exhibiting longer SP and more variability with respect to the ML direction, regardless of group, foot position, and vision condition (compare Fig. 2b and 2c for the SP, and Fig. 2e and 2f for the RMS). Significant group × direction interaction was observed for the SP (F_1,8_ = 6.78, P = 0.031, 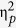
* = *0.46), with the patients showing longer SP in the AP than in ML direction compared with healthy participants (compare Fig. 2b and 2c). No effect of this interaction there was for the RMS (F_1,8_ = 0.01, P = 0.91; compare Fig. 2e and 2f).

Thus, the overall reduction of the postural stability associated with PD depends on a concomitant increase of spatial displacement and temporal variability of the COP motion. Specific increase of the COP displacement in patients with PD was detected for the AP direction.

Numerical data for the stability-related parameters are reported in the Table 3, rows 1–6 and 12–17.

### Analysis of postural adaptation to foot position and vision condition

The analysis of stability modifications associated with changes in foot position and vision condition showed a typical main scheme: the upright stability worsened passing from spaced to nearby foot position or passing from eyes open to closed. In fact, except for the SP AP and RMS AP, there was a main effect of foot position alone among all the other stability parameters (Table 2: column 2, rows 1–6), with the most instable stance (feet extra-rotated with the heels together and opening angle of 30°, FP30) showing significant differences with respect to easier stances (parallel feet with the heels spaced 20 cm, FP20 and 10 cm, FP10) (Table 2: post hoc FP, rows 1, 3, 4, 6). In the same vein, with the exception of the RMS ML, significant differences were observed between the two vision conditions across the stability-related parameters (Table 2: column 3, rows 1–6).

However, while foot position did not show interaction effects with the group for all the stability-related parameters (Table 2: column 4, rows 1–6), there were significant group × vision condition interaction over all the stability-related parameters, except for the RMS ML (Table 2: column 5, rows 1–6). The gap between eyes open and eyes closed was larger in the patients than in the control participants (Fig. 2a–e; Table 3: rows 1–5 and 12–16). Moreover, when the two directions were compared, significant group × vision × direction interaction was exhibited for the SP and RMS (SP: F_1,8_ = 6.46, P = 0.035, 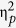
* = *0.45; RMS: F_1,8_ = 13.88, P = 0.006, 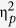
* = *0.63). These results suggest that, passing from eyes open to closed, the difference between the two directions increased more in the patients than in healthy subjects, with larger worsening in the AP than ML direction (compare Fig. 2b and 2c for SP, and Fig. 2e and 2f for RMS).

Overall, while the changes in foot position did not influence the gap of stability between the two groups, the postural performance worsened even more passing from eyes open to closed, especially along the AP oscillations.

### Analysis of the frequency domain and nonlinear spatio-temporal parameters

The parameters associated with the structure of the sway motion (Fig. 2g–k) showed no statistically significant differences for group, foot position and group × foot position interaction (Table 2: columns 1, 2, 4; rows 7–11). The only significant effects observed among the structure-related parameters were associated with the vision condition and the interaction group × vision condition: the values of the Fractal Dimension (FD), the Approximate Entropy (ApEn) in the AP direction (ApEn AP) and the Mean Power Frequency (MPF) in AP direction (MPF AP) increased passing from eyes open to closed (Table 2: column 3; rows 7, 8, 10; Fig. 2g,h,j), while group × vision condition interaction was significant for the FD, the ApEn in ML direction (ApEn ML) and the MPF in ML direction (MPF ML; Table 2, column 5; rows 7, 9, 11; Fig. 2g,i,k).

Significant influences of direction on the structure-related parameters were limited to a main effect of direction for MPF (F_1,8_ = 5.47, P = 0.048, 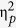
* = *0.41; Fig. 2j,k) and group × vision condition interaction for the ApEn (F_1,8_ = 6.11, P = 0.039, 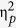
* = *0.43; Fig. 2h,i) and the MPF (F_1,8_ = 6.37, P = 0.036, 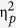
* = *0.44; Fig. 2j,k). Unlike the stability-related parameters, the frequency contribution to the COP signal and the nonlinear dynamic of the spatio-temporal COP motion are mostly similar between the two groups. Some variations of these parameters were associated with the changes in sway direction or vision condition.

The effect sizes reported for all the statistical analyses showed a good overall level of variance explained by the ANOVA factors for the significant results (Table 2). The mean effect size measured as η^2^_p_ for the stability- and structure-related parameters was 0.61, with a range between 0.40 and 0.85 for the stability parameters and between 0.46 and 0.88 for the structure-related parameters.

### Comparison of muscle strength with postural stability in each single patient

The five patients exhibited different levels of muscle strength across the lower limbs segments (Table 1). In three patients (#3-#4-#5), proximal muscles were weaker than distal muscles (MRC grade 2–3 at the hip vs MRC grade 3–4 at the ankle), whereas two patients (#1 and #2) showed normal muscle strength (grade 5) over all lower limb muscles. At the hip joint, the abductor muscles were about 1 MRC grade stronger than others.

**Figure 3:**
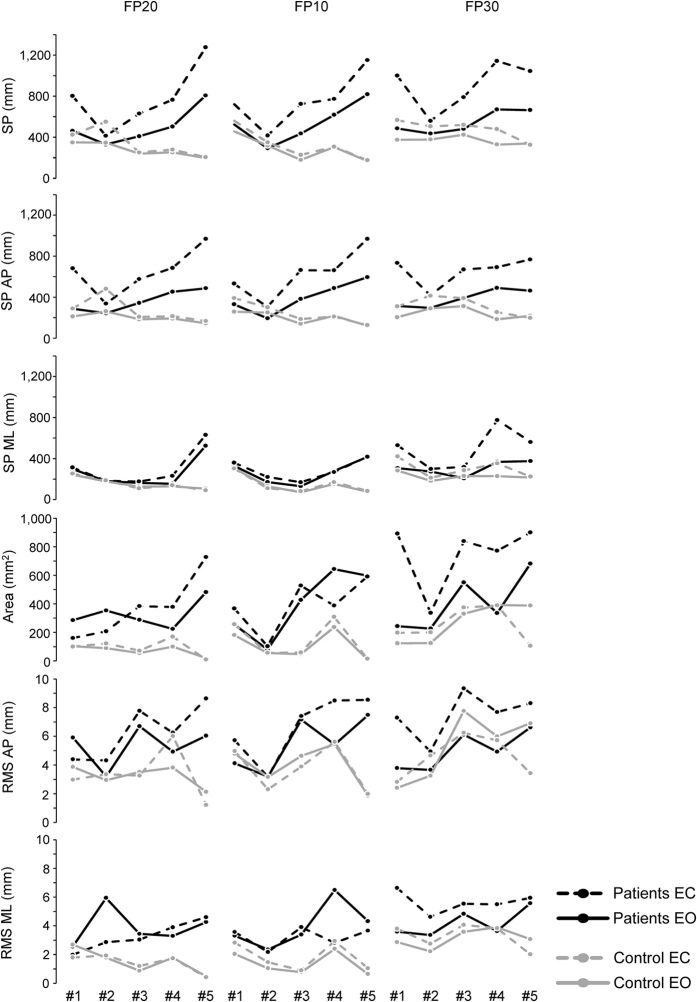
Quantitative changes in stability-related parameters for each single participant. The changes in stability-related parameters across the foot positions (FP20, FP10, FP30), and passing from eyes open (EO) to eyes closed (EC) are reported as single measurements for each of the ten participants.

Comparing these data with single measurements of the stability-related parameters obtained from each participant across the three foot positions (Fig. 3), qualitative association between the changes in the level of muscle weakness and the changes in the postural performance can be identified.

The patient #5 exhibited the lowest level of muscle force and the lowest postural stability in almost all the parameters. On the other hand, in the patients #1 and #2, an overall good level of postural performance was parallel to a normal level of muscle strength as evaluated by the MRC scale. In particular, the level of postural stability in the patient #2 was close to the results observed in the healthy subjects for almost all the parameters. Finally, the patients #3 and #4 showed intermediate levels for both muscle strength and postural stability.

**Figure 4:**
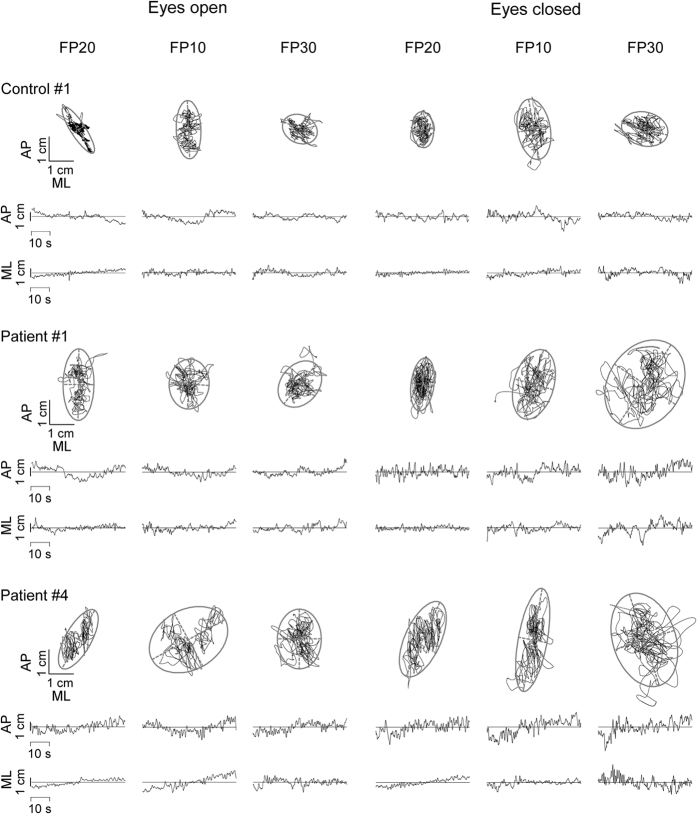
Representative examples of the center of pressure measurements. The plots illustrate the two-dimensional trajectories of the center of pressure and the temporal variability of the motion along anterior-posterior (AP) and medial-lateral (ML) directions measured in single trials for a control subject (#1) and for two patients with PD (#1 and #4).

In Fig. 4, the original data regarding the COP spatial displacement and the temporal variability of the signals along AP and ML directions are reported for the patients #1 and #4 and for an healthy participant (#1). Consistent with the overall statistical analysis, the spatial extension and the temporal variability of the COP motion were larger in the patients than in the healthy subject. In particular, along the AP direction and for FP30, the patients showed greater worsening than the healthy participant passing from eyes open to closed. In accord with more postural stability and less muscle weakness exhibited by the patient #1 with respect to the patient #4 (Fig. 3 and Table 1), the patient #4 showed larger spatial COP trajectory and temporal variability than the patient #1 (Fig. 4).

## Discussion

In this study, we adopted an instrumented quantitative approach to analyze the quiet upright standing in patients with late-onset PD. The results reported in this paper can contribute to overcome the limitation of subjective descriptions of the balance deficits associated with PD, typically provided to date by the relevant literature.

The patients with PD exhibited an increase in amplitude and variability of the sway oscillations with respect to the healthy persons. These differences were observed over different foot positions, with important worsening along AP direction and during stance with eyes closed. In the group of patients these changes were parallel to the different levels of muscle weakness. A reduced number of modifications were detected between the two groups for the frequency content and the spatio-temporal structure of the sway motion.

The weakness of antigravity muscles is the most straightforward explanation for the overall postural deficits observed in the patients with PD. The association between muscle weakness and standing impairments is reported by several authors^19^,^20^,^26^,^27^, and, in the current study, it is supported by the following two outcomes.

First, the changes in muscle strength in the lower limbs of the patients, evaluated by the MRC score, were parallel to the changes in postural stability: the patients with the lowest scores exhibited the greatest instability and vice versa.

Second, most of the differences observed between the two groups were focused on stability-related parameters, while few significant results were reported for the parameters associated with the structure of sway motion. This means that the level of complexity to accomplish the basic postural strategies was similar between the patients and the healthy participants, but the mechanical execution required a diverse strength. It is possible that patients with late-onset PD present a typical development of balance coordination, and later, at the appearance of the disease symptoms, only the level of strength is impaired. To test this hypothesis, it would be of some interest to apply the same experimental protocol to patients with early-onset PD. However, these considerations concern the standing with eyes open, since other components than muscle weakness must be taken into account when standing was performed with eyes closed (see the specific section below).

Most of the balance deficits observed in patients with PD occurred along the AP direction, as it appears comparing SP and RMS measurements for the two directions. Moreover, a consistent postural stability was maintained in the ML direction also when the medial-lateral axis of the feet surface decreased passing from 20 cm (FP20) to 10 cm (FP10) inter-foot distance. On the other hand, when the AP axis was reduced, as in the position with the feet 30 degree rotated (FP30), the largest COP motion among the three foot configurations was observed.

Generally, in healthy subjects the sway motion along the AP component of the COP is more instable than the sway in the ML component during both quiet^28^ and perturbed stance^29^,^30^. Greater instability along AP direction depends on the passive mechanical constrains at ankle and knee joints. In fact, the anatomical conformation of these joints largely minimizes the ML motion, while more degrees of freedom are available at the hip joint^28^. Thus, the muscles serving the distal joints produce sway movements along the AP direction, while the muscular activity at the hip is aimed to control both AP and ML directions^30^,^31^. Noteworthy, passive constrains influence postural stability mainly during the static upright stance^28^.

On these bases, the additional reduction in motion along the AP direction observed in the patients can be explained by the fact that muscle weakness may produce more impairments along the AP than ML direction as most of the perturbations along the ML direction are compensated by the intrinsic passive joint constraints. This interpretation is supported by the findings of Horlings *et al.*^20^. These authors compared postural reactions to platform rotations of healthy subjects with respect to patients with muscle weakness caused by neuromuscular disorders. As in the current study, also under dynamic balance condition the patients showed more instability for the sway motion along the AP direction (pitch rotation) than for the motion in the ML direction (roll rotation).

Proximal muscles (trunk and hip joints muscles) could give a special contribution to the balance deterioration in the patients with PD. In fact, a non-uniform distribution of the level of force over the muscular apparatus was reported in patients with late-onset PD, with the proximal muscles more impaired than the distal muscles^1^,^3^,^6^,^8^,^9^. The MRC strength scores reported in Table 1 confirm this trend for three out of the five patients, with the MRC grade increasing from the hip to knee up to ankle muscles. Considering that the flexor-extensor muscles produce the body sway along the AP direction, while the abductor-adductor muscles influence the movements in the ML direction, the results of the MRC evaluation are in accord with the larger instability observed in AP with respect to the ML direction. In fact, at the hip, the flexors exhibited less strength than the abductor muscles. In addition, the flexor-extensor muscles are more prominent than the abductor-adductor muscles across the lower limbs. Thus, it is reasonable to assume that the asymmetric distribution and the different force production of the flexor-extensor and abductor-adductor muscles might have played an important role in determining the overall asymmetric stability reported in the current paper.

When studying the factors that influence standing stability, it should be considered that postural perturbations might also originate from the body itself. For example, voluntary limbs movements are the main source for the internal perturbations of upright posture^32^,^33^. Similarly, involuntary mechanical changes in the cardio-vascular and respiratory systems may influence the standing posture, particularly during static condition^34^,^35^,^36^. Important impairments of respiratory function can be observed in patients with PD due to respiratory muscle weakness^7^,^10^,^11^. Although we did not perform direct measurements of respiratory function, this factor can be considered as a potential contributor to the balance defects observed in this work.

As the central nervous system prevents upright standing perturbations that arise from voluntary movements by accomplishing anticipated postural adjustments^32^,^33^, similarly, a predictive control structure may produce novel muscle synergies at the trunk and lower limbs and compensate for respiratory perturbations to posture^37^,^38^.

This compensation could be absent in diseases with breathing cycle impairment. For example, Grimstone and Hodges^18^ found a reduced postural compensation for respiratory perturbations in patients suffering of back pain. The considerable respiratory deficits in patients with late-onset PD could justify the absence of postural compensation, and thus the negative influence on upright standing.

A signal supporting the lack of postural adaptation associated with breathing impairment in PD might be the limited variations of the structure-related parameters between the two groups observed in the current study. In fact, although we cannot evaluate the level of sensibility of these parameters, the emerging of novel compensatory strategies should determinate changes in frequency domain and/or in the spatio-temporal structure of sway motion.

A possible postural adaptation emerging from our data regards the standing behavior associated with the foot position changes from easy to more difficult configurations. Although people with PD showed greater instability than the control group, the variations across the three foot positions were consistent between the two groups. This compensation could depend on the level of muscle force adaptation, since the effect of structure-related parameters was irrelevant for the interaction between foot positions and groups. It is possible that the level of muscle weakness characterizing our sample of patients did not prevent postural compensations for changes in foot position when the stances are restricted within quiet and static postures.

On the contrary, an important reduction of the postural performance was associated with the vision condition. Maintaining upright stance relies on the integration of vision, vestibular and proprioceptive sensory signals^39^,^40^. In the current study, when the participants performed the postural tests with eyes closed, a moderate stability reduction was observed in the healthy subjects^39^, whereas the patients showed a much stronger deterioration of postural stability, especially in the AP direction.

Although muscle weakness may be responsible for the further reducing in standing stability during stances with eyes closed in patients with PD, we believe that a suitable interpretation of this outcome could be based on the complex interaction of the sensory channels providing information to the postural control system.

Several data suggest that a reduction of visual accuracy produces less postural instability than inaccurate proprioceptive information^39^,^41^. However, each sensory channel can change the relative contribution to postural control, following an integration process aimed to reweight the single inputs in relation to the environment demand^39^,^42^ or with age and diseases^43^,^44^. For example, when healthy subjects close their eyes, a reweighting of proprioceptive and vestibular signals can compensate for the reduction in visual information^42^. On the same vein, older people with glaucoma rely more on vestibular and proprioceptive signals to keep upright standing, and the balance stability decrease when vestibular or proprioceptive sensory signal was affected^43^.

Overall, the reduction in balance stability associated with the visual condition in patients with PD might reflect a failure in sensory reweighting. In other words, possible proprioceptive and/or vestibular deficits in patients with PD may prevent an appropriate sensory integration to compensate for the lack of visual information.

Although the information on the contribution of nervous dysfunctions to the clinical features of PD are sparse^7^, the histological evidences of abnormal muscle spindles in the muscles of patients with late-onset PD^6^, and the presence of glycogen accumulation in the peripheral nerves and spinal ganglia^12^,^13^,^15^, indicate the possibility that proprioceptive information is impaired in PD, supporting our hypothesis of partial or no compensation for visual inaccuracy. This suggestion is reinforced by the significant interaction observed between group and vision condition for the structure-related parameters. A reorganization of sensory information may determine variations in the complexity of sensory integration, producing the observed differences between the groups in frequency content and in the spatio-temporal structure of the signal.

More in general, these findings suggest a reconsideration of neuronal factors influencing gross motor function, such as upright standing or gait movements, in patients with PD^25^. In fact, the complex elaboration accomplished by the nervous system to control these motor tasks requires the integrity of both sensory inputs and motor output. Several changes in central and motor output elements have been associated with PD^4^,^5^,^7^,^12^,^13^,^14^,^17^, but specific investigations on deficits of the sensory system may help to detect with more accuracy the underlying causes affecting many of the everyday motor skills in patients with PD.

The restricted sample size, due to the rarity of Pompe disease, is a limitation to the study design. However, the good level of effect sizes corroborates the reliability of the results observed here. In addition, in Fig. 3 we provided the original measurements for each single participant to facilitate a more direct inspection of the variability and the differences between the two groups.

We recognize that an objective quantification of muscle force and an evaluation of the respiratory function would have been important for the completeness of the study. However, the main objective of this study was to quantify with an objective approach the impact of PD on the upright posture. Future studies on more specific functional and clinical relationships may benefit from the data provided by this paper.

## Conclusions

Quantitative analysis of quiet upright standing in patients with PD reveals an overall reduction of the postural stability without changes in the structure of the sway motion. Specific balance impairments associated with the sway direction and with the availability of visual information are discussed in relation to the topographical distribution of muscle weakness, respiratory insufficiencies and proprioceptive input deficits.

This study strongly suggests to consider the sensory impairment, poorly documented in the specialized literature on PD, as a potential element affecting the motor performance, especially for multi-segments tasks that require a complex sensory-motor integration.

The data of the current work can serve as a base for further investigations on the effects of PD on upright posture and other everyday motor skills. Moreover, we deemed that these results might help to monitor the disease evolution and to improve the accuracy of clinical interventions.

## Material and Methods

### Subjects

Ten male participants took part in the study. Five patients with confirmed diagnosis of PD (age, 29.6 ± 12.4 years; height, 182.3 ± 10.7 cm; weight, 82.7 ± 15.8 kg; Table 1) were compared with a control group of five age matched healthy participants (age, 32.8 ± 11.3 years; height, 180.6 ± 5.0 cm; weight, 77.1 ± 7.0 kg). Age, height and weight did not significantly differ between the groups (age, P = 0.68; height, P = 0.76; weight, P = 0.49). The limited sample size is due to the rarity of the Pompe disease. All the patients were treated with enzyme replacement therapy (ERT; Myozyme, mean dose, 20 mg/kg, every 2 weeks by I.V. infusion at the hospital) for their clinical needs. They were able to walk independently. The study protocol was approved by the Ethical Board of the University Hospital of Catania. All the subjects gave informed written consent, and the methods were carried out in accordance with the Declaration of Helsinki.

### Apparatus and procedures

A force platform (KISTLER 9286 B, Winterthur, CH; 200 Hz sampling frequency) recorded the reactive forces during the balance tests and sent the signals to the SMART-D system (BTS, Garbagnate Milanese, MI, IT) for offline processing.

Experimental sessions were performed two days before the ERT. Preliminary physical examination allowed to collect anthropometric measures (height, weight) and range of motion and muscle strength at the hip, knee and ankle joints. Muscles strength was evaluated by using the MRC scale^45^. This categorical scale was proven to be a reliable tool to evaluate volitional muscle strength in cooperative patients^46^.

After the physical examination, the participants received adequate explanation concerning the procedure to follow. The postural assessment protocol consisted to perform a sequence of quiet stances with the foot positions configured as follow (Fig. 1):

1. parallel feet with the heels spaced 20 cm (FP20);
2. parallel feet with the heels spaced 10 cm (FP10);
3. feet extra-rotated with the heels together and opening angle of 30° (FP30).

These configurations imply a progressive reduction of the postural stability, since the base of support decreases from FP20 to FP30 (Fig. 1). Moreover, passing from FP20 to FP30, the instability increases more along the ML than AP direction.

For each foot position, the test was performed with eyes open and closed. The order of presentation of combinations of foot position and vision condition was randomized across the participants.

The subjects stood barefoot with their arms placed downward at their sides of the body, and their eyes focusing on a mark placed at a distance of 2.5 m. The feet were placed inside an outline borders to guarantee a consistent foot positioning across the tests. The participants were asked to keep the upright posture as immobile as possible for 50 sec, with an inter-trial interval of 2 min.

To prevent fatigue, the participants were instructed to alert the experimenters if they were feeling fatigue, and the experimenters occasionally asked the participants about their fatigue level. However, given the low level of physical performance, and the large fraction of time for the rest (10 min) with respect to the time for the tests (5 min), fatigue was never an issue.

The sessions took place in a room at the temperature of 22–24 °C, without external noises and with diffused light.

### Data processing and measurements

Raw data were low-pass filtered using a zero-lag second-order Butterworth filter with 5-Hz cutoff frequency.

The AP and ML coordinates of the COP position were computed from forces and torques values measured by the force platform and elaborated offline by the software Sway (BTS Garbagnate Milanese MI, IT). From the AP and ML time series, the two-dimensional trajectory of the COP was reconstructed, and two sets of parameters were obtained.

The first set of parameters describes the level of stability of the COP (stability-related parameters):

Area: total area covered by the COP trajectory computed as the 95% confidence ellipse;

SP: the total length of the COP trajectory computed as the sum of the distances between two consecutive points in the two-dimensional space;

SP AP and SP ML: the length of the COP displacement computed as SP, but measured along the AP and ML directions;

RMS AP and RMS ML: variability along the AP and ML directions computed as standard deviation from the mean of each time series.

The second set of parameters describes the dynamical structure of postural signals in time, space and frequency domain (structure-related parameters):

ApEn AP and ApEn-ML: estimation of the level of regularity of oscillations along the AP and ML directions, taking into account the non-stationarity property of the postural signal. The ApEn was computed using input parameters based on our data and former established protocols^47^,^48^: the time series length was 10,000 points; the pattern length of compared data was 2 data points; the tolerance window was normalized to 0.2 times the standard deviation of individual time series; the lag value was set to 10. The ApEn ranges between 0 and 2 with 0 indicating a linear phenomenon with high regularity, while 2 indicating a data behavior completely random.

FD: a measure of the two-dimensional COP trajectory complexity. The FD was computed using the following equation^49^:


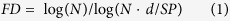


where *N* is the number of data points (*N* = 10,000); *d* = (2*a* · 2*b*)^1/2^ where *a* and *b* are the major and minor axes of the 95% confidence ellipse, respectively. The geometrical complexity of COP trajectory increases as the two-dimensional FD passes from 0 to 2.

MPF AP and MPF ML: represents the mean frequency contained within a power spectrum, and was determined for AP and ML directions as follow:


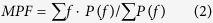


where *f* represents frequencies in the signal, and *P* is the amplitude of Power Spectral Density (PSD) at each frequency. The PSD was computed from unfiltered AP and ML time series using the multitaper estimation method^49^. Since no discernable spectral peaks were visible above 1.5 Hz, frequency domain measures were calculated in the range 0.025–1.5 Hz (bins of 0.025 Hz). The first bin past the dc component was not included in the analysis.

All the computations were performed using a customized MatLab code (MatLab R2012a, Mathworks, Natick, MA, USA).

### Statistical analysis

Preliminary tests for normality (Shapiro-Wilk test) and for equality of sample variances (Levene’s test) were performed to provide the basis for using parametric statistics on a small sample.

For each condition, each parameter was quantified computing mean, standard deviation, standard error and range over all the five participants. Since the small sample, range was used as measure of variability in the analytic summary of descriptive statistics, whereas, for clarity of illustration, standard error was used as error bars in the plots.

For repeated measures ANOVA (see Results section), the critical value of F was adjusted applying Greenhouse-Geisser correction, that produces a P-value more conservative. This procedure corrects the repeated measures ANOVA with respect to a possible violation of the sphericity assumption, that is, the variance of the differences among all combinations of independent variables must be equal.

The level of significance was set at P < 0.05 for all the statistical tests. Multiple post hoc pairwise comparison was performed by two-tailed paired t-test with Bonferroni correction.

To assess the magnitude of the ANOVA outcomes, the effect sizes were estimated by using partial eta squared values (η^2^_p_). The η^2^_p_ describes the percentage of variance of the dependent variable attributed to the independent variables of interest. For the correlated samples in the post hoc paired test, the effect sizes were determined by using Hedges’ g_av_, which standardized mean differences based on the average standard deviation of both repeated measures. The Hedges’ g represents a correction of the more common Cohen’s d index, since the latter provides a biased estimate of the population effect size. This correction is particularly crucial when small samples are compared. The effect size computations were based on the recommendation suggested by Laken^50^.

Statistical analysis was performed using Systat 11 (Systat Inc., Evanston, IL, USA).

## Additional Information

**How to cite this article**: Valle, M. S. *et al.* Quantitative analysis of upright standing in adults with late-onset Pompe disease. *Sci. Rep.* **6**, 37040; doi: 10.1038/srep37040 (2016).

**Publisher’s note:** Springer Nature remains neutral with regard to jurisdictional claims in published maps and institutional affiliations.

## Figures and Tables

**Table 1:** Anthropometric and clinical characteristics of the five study patients.

Patients	Age at postural test (years)	Time between 1^st^ ERT and postural test (years)	Height (cm)	Weight (kg)	Hip Strength		Knee Strength	Ankle Strength
					MRC (0–5) Flex/Ext	MRC (0–5) Abd/Add	MRC (0–5) Flex/Ext	MRC (0–5) Flex/Ext
#1 (AP)	18	8	187	69	5/5 (R)	5/5 (R)	5/5 (R)	5/5 (R)
					5/5 (L)	5/5 (L)	5/5 (L)	5/5 (L)
#2 (RG)	20	6	193	78	5/5 (R)	5/5 (R)	5/5 (R)	5/5 (R)
					5/5 (L)	5/5 (L)	5/5 (L)	5/5 (L)
#3 (DD)	26	9	188	94	4/4 (R)	4/4 (R)	4/4 (R)	4/4 (R)
					3/4 (L)	4/4 (L)	4/4 (L)	4/4 (L)
#4 (AC)	36	5	166	99	3/3 (R)	4/3 (R)	4/4 (R)	4/4 (R)
					3/3 (L)	4/3 (L)	4/4 (L)	4/4 (L)
#5 (AT)	48	4	177	65.4	2/2 (R)	3/2 (R)	3/3 (R)	4/4 (R)
					2/2 (L)	3/2 (L)	3/3 (L)	4/4 (L)

ERT: Enzyme Replacement Therapy; MRC: Medical Research Council scale; Flex: Flexor; Ext: Extensor; Abd: Abductor; Add: Adductor; R: Right; L: Left.

**Table 2:** Three-way repeated measures ANOVA summarizing the overall postural changes.

Parameters		1. G	2. FP	post hoc FP			3. VC	4. G x FP	5. G x VC
				10/20	10/30	20/30			
		df: 1, 8	df: 2, 16				df: 1, 8	df: 2, 16	df: 1, 8
1. SP	P	**0.011**	**0.023**	0.896	**0.024**	**0.039**	**<0.001**	0.782	**0.004**
	F	10.76	5.86				44.15	0.17	15.86
	ES	0.57	0.42		0.56	0.53	0.85		0.66
2. SP AP	P	**0.006**	0.321				**<0.001**	0.676	**0.007**
	F	13.69	1.22				34.95	0.4	12.99
	ES	0.63					0.81		0.62
3. SP ML	P	0.062	**0.009**	0.798	**0.002**	**0.025**	**0.002**	0.938	**0.039**
	F	4.72	9.32				19.88	0.08	6.03
	ES		0.54		1.21	1.05	0.71		0.43
4. AREA	P	**0.007**	**<0.001**	0.245	**0.002**	**0.001**	**0.016**	0.797	**0.036**
	F	12.76	16.08				9.23	0.23	6.33
	ES	0.61	0.67		0.91	1.27	0.54		0.44
5. RMS AP	P	**0.048**	0.059				**0.027**	0.369	**0.005**
	F	5.45	3.38				7.26	1.06	14.74
	ES	0.40					0.48		0.65
6. RMS ML	P	**<0.001**	**<0.001**	0.795	**<0.001**	**0.002**	0.522	0.801	0.639
	F	30.45	16.10				0.45	0.23	0.24
	ES	0.79	0.67		1.44	1.54			
7. FD	P	0.847	0.053				**<0.001**	0.355	**0.032**
	F	0.04	3.55				56.31	1.11	6.72
	ES						0.88		0.46
8. ApEn AP	P	0.243	0.155				**0.003**	0.518	0.95
	F	1.59	2.10				17.88	0.68	0.00
	ES						0.69		
9. ApEn ML	P	0.269	0.24				0.128	0.366	**0.023**
	F	1.46	1.56				2.88	1.07	7.90
	ES								0.50
10. MPF AP	P	0.204	0.071				**0.005**	0.371	0.989
	F	1.91	3.16				14.57	1.06	0.00
	ES						0.65		
11. MPF ML	P	0.379	0.301				0.383	0.448	**0.023**
	F	0.87	1.29				0.85	0.84	7.92
	ES								0.50

G: Group; FP: Foot Position; VC: Vision condition; df: degree of freedom, P: probability value; F: F statistic; ES: Effect Size. Significant values are indicated in bold. The effect sizes are expressed as partial eta squared (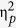) values for the ANOVA factors and as Hedges’ g_av_ index for the post hoc comparison.

**Table 3:** Summary of descriptive statistics for all the parameters and experimental conditions.

Parameters	Eyes open					
	FP20		FP10		FP30	
	controls (n = 5)	patients (n = 5)	controls (n = 5)	patients (n = 5)	controls (n = 5)	patients (n = 5)
1. SP (mm)	279 (199–351)	504 (332–807)	282 (165–454)	535 (289–816)	373 (332–428)	551 (439–674)
2. SP AP (mm)	199 (146–261)	363 (244–487)	204 (128–264)	403 (200–600)	247 (190–317)	396 (300–495)
3. SP ML (mm)	156 (106–243)	264 (154–525)	149 (73–307)	267 (133–418)	227 (182–282)	306 (207–375)
4. AREA (mm^2^)	73 (19–103)	326 (223–481)	113 (19–241)	404 (86–645)	278 (130–397)	413 (233–686)
5. RMS AP (mm)	3.3 (2.1–3.9)	5.3 (3.2–6.7)	4.0 (1.9–5.5)	5.5 (3.2–7.5)	5.3 (2.4–7.8)	5.1 (3.7–6.7)
6. RMS ML (mm)	1.5 (0.5–2.7)	3.9 (2.6–5.9)	1.4 (0.7–2.4)	4.0 (2.4–6.5)	3.1 (2.2–3.9)	4.2 (3.4–5.6)
7. FD	1.59 (1.51–1.67)	1.53 (1.43–1.61)	1.55 (1.45–1.63)	1.54 (1.46–1.58)	1.50 (1.41–1.58)	1.54 (1.46–1.61)
8. ApEn AP*	2.3 (1.8–3.3)	2.3 (1.8–3.5)	2.0 (1.1–3.0)	2.7 (1.9–3.4)	2.1 (1.1–3.4)	3.0 (2.3–3.7)
9. ApEn ML*	4.9 (2.6–9.0)	2.8 (1.1–5.0)	4.3 (2.4–6.0)	2.7 (1.4–3.9)	2.8 (2.2–3.7)	2.8 (1.5–3.9)
10. MPF AP (Hz)*	8.3 (6.6–11.2)	9.2 (5.6–12.8)	6.3 (4.6–9.9)	9.9 (6.1–13.2)	7.0 (3.9–12.1)	10.7 (7.5–13.9)
11. MPF ML (Hz)*	18.7 (9.9–37.0)	10.3 (3.9–21.9)	15.0 (8.0–22.3)	10.2 (5.5–16.7)	10.4 (7.9–13.1)	10.3 (4.9–14.9)
	**Eyes closed**					
	**FP20**		**FP10**		**FP30**	
12. SP (mm)	344 (207–552)	778 (416–1276)	321 (172–556)	754 (414–1148)	483 (330–570)	911 (562–1147)
13. SP AP (mm)	273 (169–481)	650 (337–968)	248 (133–396)	631 (312–973)	318 (203–419)	661 (422–772)
14. SP ML (mm)	155 (90–253)	307 (176–630)	152 (84–305)	289 (171–419)	299 (213–420)	496 (299–774)
15. AREA (mm^2^)	94 (9–169)	371 (160–727)	145 (22–313)	399 (109–594)	259 (113–395)	751 (342–902)
16. RMS AP (mm)	3.4 (1.22–6.01)	6.3 (4.3–8.6)	3.8 (2.0–5.7)	6.7 (3.2–8.6)	4.6 (2.9–6.3)	7.6 (5.0–9.4)
17. RMS ML (mm)	1.4 (0.4–1.9)	3.3 (2.0–4.6)	1.8 (0.9–2.9)	3.2 (2.2–3.9)	3.3 (2.0–4.1)	5.7 (4.6–6.7)
18. FD	1.64 (1.47–1.80)	1.64 (1.54–1.78)	1.56 (1.42–1.66)	1.62 (1.56–1.68)	1.56 (1.50–1.63)	1.58 (1.53–1.64)
19. ApEn AP*	3.7 (1.3–5.7)	4.1 (2.7–6.2)	2.7 (1.4–5.2)	3.6 (2.9–4.4)	2.7 (1.6–4.2)	3.3 (2.6–3.8)
20. ApEn ML*	4.8 (2.9–8.5)	3.6 (2.1–6.3)	3.1 (2.1–4.2)	3.4 (1.6–4.3)	3.5 (2.6–4.3)	3.3 (2.0–5.6)
21. MPF AP (Hz)*	14.5 (4.9–23.4)	15.6 (8.0–24.8)	9.4 (4.7–20.0)	13.7 (9.2–21.3)	8.7 (5.9–12.2)	11.4 (8.0–12.9)
22. MPF ML (Hz)*	16.9 (11.5–29.0)	13.6 (6.2–24.5)	10.6 (8.2–14.4)	13.3 (5.8–17.9)	12.4 (9.9–16.5)	11.9 (6.6–20.3)

Data are expressed as mean and range. The range is indicated in parenthesis. Asterisks (*) indicate rows with numbers (*n*) expressed by the following exponential notation: *n* ×10^2^.

## References

[b1] Hirschhorn R. & Reuser A. J. Glycogen storage disease type II; acid alpha-glucosidase (acid maltase) deficiency in The metabolic and molecular bases of inherited disease 8th edn (eds Scriver C. R., Beaudet A. L., Sly W.& Valle D.) 3389–3420 (McGraw-Hill, 2001).

[b2] Kishnani P. S. *et al.* Pompe disease diagnosis and management guideline. Genet. Med. 8, 267–288 (2006). 16702877 10.1097/01.gim.0000218152.87434.f3 PMC3110959

[b3] Bembi B. *et al.* Diagnosis of glycogenosis type II. Neurology 71, S4–11 (2008). 19047572 10.1212/WNL.0b013e31818da91e

[b4] Hogan G. R., Gutmann L., Schmidt R. & Gilbert E. Pompe’s disease. Neurology 19, 894–900 (1969). 5257391 10.1212/wnl.19.9.894

[b5] DiMauro S., Stern L. Z., Mehler M., Nagle R. B. & Payne C. Adult-onset acid maltase deficiency: a postmortem study. Muscle Nerve 1, 27–36 (1978). 377069 10.1002/mus.880010105

[b6] van der Walt J. D., Swash M., Leake J. & Cox E. L. The pattern of involvement of adult-onset acid maltase deficiency at autopsy. Muscle Nerve 10, 272–281, doi: 10.1002/mus.880100311 (1987). 2951596

[b7] Fuller D. D. *et al.* The respiratory neuromuscular system in Pompe disease. Respir. Physiol. Neurobiol. 189, 241–249, doi: 10.1016/j.resp.2013.06.007 (2013). 23797185 PMC4083814

[b8] Musumeci O. *et al.* Homozygosity for the common GAA gene splice site mutation c.-32-13T > G in Pompe disease is associated with the classical adult phenotypical spectrum. Neuromuscul. Disord. 25, 719–724, doi: 10.1016/j.nmd.2015.07.002 (2015). 26231297

[b9] Kassardjian C. D., Engel A. G. & Sorenson E. J. Electromyographic findings in 37 patients with adult-onset acid maltase deficiency. Muscle Nerve 51, 759–761 (2015). 25703805 10.1002/mus.24620

[b10] Smith B. K., Corti M., Martin A. D., Fuller D. D. & Byrne B. J. Altered activation of the diaphragm in late-onset Pompe disease. Respir. Physiol. Neurobiol. 222, 11–15, doi: 10.1016/j.resp.2015.11.013 (2016). 26612101 PMC4869700

[b11] Pellegrini N. *et al.* Respiratory insufficiency and limb muscle weakness in adults with Pompe’s disease. Eur. Respir. J. 26, 1024–1031, doi: 10.1183/09031936.05.00020005 (2005). 16319331

[b12] Gambetti P., DiMauro S. & Baker L. Nervous system in Pompe’s disease. Ultrastructure and biochemistry. J. Neuropathol. Exp. Neurol. 30, 412–430 (1971). 5284681 10.1097/00005072-197107000-00008

[b13] Martin J. J., de Barsy T., van Hoof F. & Palladini G. Pompe’s disease: an inborn lysosomal disorder with storage of glycogen. A study of brain and striated muscle. Acta Neuropathol. 23, 229–244 (1973). 4511788 10.1007/BF00687878

[b14] Teng Y. T., Su W. J., Hou J. W. & Huang S. F. Infantile-onset glycogen storage disease type II (Pompe disease): report of a case with genetic diagnosis and pathological findings. Chang. Gung. Med. J. 27, 379–384 (2004). 15366815

[b15] Sidman R. L. *et al.* Temporal neuropathologic and behavioral phenotype of 6neo/6neo Pompe disease mice. J. Neuropathol. Exp. Neurol. 67, 803–818 (2008). 18648322 10.1097/NEN.0b013e3181815994 PMC2743262

[b16] DeRuisseau L. R. *et al.* Neural deficits contribute to respiratory insufficiency in Pompe disease. Proc. Natl. Acad. Sci. USA 106, 9419–9424, doi: 10.1073/pnas.0902534106 (2009). 19474295 PMC2695054

[b17] Corti M. *et al.* Altered activation of the tibialis anterior in individuals with Pompe disease: Implications for motor unit dysfunction. Muscle Nerve 51, 877–883, doi: 10.1002/mus.24444 (2015). 25186912 PMC4348349

[b18] Grimstone S. K. & Hodges P. W. Impaired postural compensation for respiration in people with recurrent low back pain. Exp. Brain Res. 151, 218–224 (2003). 12759796 10.1007/s00221-003-1433-5

[b19] Horlings C. G. C., van Engelen B. G. M., Allum J. H. J. & Bloem B. R. A weak balance: the contribution of muscle weakness to postural instability and falls. Nat. Clin. Prac. Neurol. 4, 504–515 (2008). 10.1038/ncpneuro0886 18711425

[b20] Horlings C. G. C. *et al.* Balance control in patients with distal versus proximal muscle weakness. Neuroscience 164, 1876–1886 (2009). 19796669 10.1016/j.neuroscience.2009.09.063

[b21] Terzis G. *et al.* Effects of exercise training during infusion on late-onset Pompe disease patients receiving enzyme replacement therapy. Mol. Genet. Metab. 107, 669–673, doi: 10.1016/j.ymgme.2012.10.020 (2012). 23146291

[b22] Angelini C. *et al.* New motor outcome function measures in evaluation of late-onset Pompe disease before and after enzyme replacement therapy. Muscle Nerve 45, 831–834, doi: 10.1002/mus.23340 (2012). 22581536

[b23] Hagemans M. L. C. *et al.* Clinical manifestation and natural course of late-onset Pompe’s disease in 54 Dutch patients. Brain 128, 671–677, doi: 10.1093/brain/awh384 (2005). 15659425

[b24] van Capelle C. I. *et al.* The quick motor function test: a new tool to rate clinical severity and motor function in Pompe patients. J. Inherit. Metab. Dis. 35, 317–323, doi: 10.1007/s10545-011-9388-3 (2012). 21912959 PMC3278629

[b25] McIntosh P. T., Case L. E., Chan J. M., Austin S. L. & Kishnani P. Characterization of gait in late onset Pompe disease. Mol. Genet. Metab. 116, 152–156, doi: 10.1016/j.ymgme.2015.09.001 (2015). 26372341

[b26] Orr R. Contribution of muscle weakness to postural instability in the elderly. A systematic review. Eur. J. Phys. Rehabil. Med. 46, 183–220 (2010). 20485224

[b27] Kaya P., Alemdaroğlu İ., Yılmaz Ö., Karaduman A. & Topaloğlu H. Effect of muscle weakness distribution on balance in neuromuscular disease. Pediatr. Int. 57, 92–97 (2015). 24978611 10.1111/ped.12428

[b28] Winter D. A., Prince F., Frank J. S., Powell C. & Zabjek K. F. Unified theory regarding A/P and M/L balance in quiet stance. J. Neurophysiol. 75, 2334–2343 (1996). 8793746 10.1152/jn.1996.75.6.2334

[b29] Valle M. S., Casabona A., Cavallaro C., Castorina G. & Cioni M. Learning Upright Standing on a Multiaxial Balance Board. PLoS One 10, e0142423, doi: 10.1371/journal.pone.0142423 (2015). 26544694 PMC4636294

[b30] Carpenter M. G., Allum J. H. & Honegger F. Directional sensitivity of stretch reflexes and balance corrections for normal subjects in the roll and pitch planes. Exp. Brain Res. 129, 93–113 (1999). 10550507 10.1007/s002210050940

[b31] Moore S. P., Rushmer D. S., Windus S. L. & Nashner L. M. Human automatic postural responses: responses to horizontal perturbations of stance in multiple directions. Exp. Brain Res. 73, 648–658 (1988). 3224674 10.1007/BF00406624

[b32] Hodges P. W., Cresswell A. G. & Thorstensson A. Preparatory trunk motion accompanies rapid upper limb movement. Exp. Brain Res. 124, 69–79 (1999). 9928791 10.1007/s002210050601

[b33] Aimola E., Santello M., La Grua G. & Casabona A. Anticipatory postural adjustments in reach-to-grasp: effect of object mass predictability. Neurosci. Lett. 502, 84–88, doi: 10.1016/j.neulet.2011.07.027 (2011). 21810452

[b34] Jeong B. Y. Respiration effect on standing balance. Arch. Phys. Med. Rehabil. 72, 642–645 (1991). 1859257

[b35] Caron O., Fontanari P., Cremieux J. & Joulia F. Effects of ventilation on body sway during human standing. Neurosci. Lett. 366, 6–9 (2004). 15265579 10.1016/j.neulet.2004.04.085

[b36] Hamaoui A. *et al.* Postural disturbances resulting from unilateral and bilateral diaphragm contractions: a phrenic nerve stimulation study. J. Appl. Physiol. 117, 825–832, doi: 10.1152/japplphysiol.00369.2014 (2014). 25150226

[b37] Gurfinkel V. S., Kots Y. M., Paltsev E. I. & Feldman A. G. The compensation of respiratory disturbances of erect posture of man as an example of the organisation of interarticular interaction in Models of the structural functional organisation of certain biological systems (eds Gelfand I. M., Gurfinkel V. S., Formin S. V., Tsetlin M. L.) 382–395 (MIT Press, 1971).

[b38] Hodges P. W., Gurfinkel V. S., Brumagne S., Smith T. C. & Cordo P. C. Coexistence of stability and mobility in postural control: evidence from postural compensation for respiration. Exp. Brain Res. 144, 293–302 (2002). 12021811 10.1007/s00221-002-1040-x

[b39] Peterka R. J. Sensorimotor integration in human postural control. J. Neurophysiol. 88, 1097–1118 (2002). 12205132 10.1152/jn.2002.88.3.1097

[b40] Maurer C., Mergner T. & Peterka R. J. Multisensory control of human upright stance. Exp. Brain Res. 171, 231–250 (2006). 16307252 10.1007/s00221-005-0256-y

[b41] Clark S. & Riley M. A. Multisensory information for postural control: sway-referencing gain shapes center of pressure variability and temporal dynamics. Exp. Brain Res. 176, 299–310 (2007). 16874512 10.1007/s00221-006-0620-6

[b42] Peterka R. J. & Loughlin P. J. Dynamic regulation of sensorimotor integration in human postural control. J. Neurophysiol. 91, 410–423 (2004). 13679407 10.1152/jn.00516.2003

[b43] Kotecha A. *et al.* Balance control in glaucoma. Invest. Ophthalmol. Vis. Sci. 53, 7795–7801, doi: 10.1167/iovs.12-10866 (2012). 23060145

[b44] Pasma J. H. *et al.* Changes in sensory reweighting of proprioceptive information during standing balance with age and disease. J. Neurophysiol. 114, 3220–3233, doi: 10.1152/jn.00414 (2015). 26424578 PMC4686291

[b45] Medical Research Council. Aids to the examination of the peripheral nervous system, Memorandum no. 45, Her Majesty’s Stationery Office, London (1981).

[b46] Vanpee G., Hermans G., Segers J. & Gosselink R. Assessment of limb muscle strength in critically ill patients: a systematic review. Crit. Care Med. 42, 701–711, doi: 10.1097/CCM.0000000000000030 (2014). 24201180

[b47] Pincus S. M. Approximate entropy as a measure of system complexity. Proc. Natl. Acad. Sci. 88, 2297–2301 (1991). 11607165 10.1073/pnas.88.6.2297 PMC51218

[b48] Cavanaugh J. T. *et al.* Recovery of postural control after cerebral concussion: new insights using approximate entropy. J. Athl. Train. 41, 305–313 (2006). 17043699 PMC1569549

[b49] Prieto T. E., Myklebust J. B., Hoffmann R. G., Lovett E. G. & Myklebust B. M. Measures of postural steadiness: differences between healthy young and elderly adults. IEEE Trans. Biomed. Eng. 43, 956–966 (1996). 9214811 10.1109/10.532130

[b50] Lakens D. Calculating and reporting effect sizes to facilitate cumulative science: a practical primer for t-tests and ANOVAs. Front. Psychol. 4, 863, doi: 10.3389/fpsyg.2013.00863 (2013). 24324449 PMC3840331

